# Label-Free Analysis of Urine Samples with In-Flow Digital Holographic Microscopy

**DOI:** 10.3390/bios13080789

**Published:** 2023-08-04

**Authors:** Lucia Gigli, Nicoletta Braidotti, Maria Augusta do R. B. F. Lima, Catalin Dacian Ciubotaru, Dan Cojoc

**Affiliations:** 1 Alifax s.r.l. Via Merano, 30, Nimis, 33045 Udine, Italy; lucia.gigli@alifax.com; 2 Consiglio Nazionale Delle Ricerche (CNR), Istituto Officina dei Materiali (IOM), Area Science Park-Basovizza, Strada Statale 14, Km 163,5, 34149 Trieste, Italy; nicoletta.braidotti@phd.units.it (N.B.); ma.rbfl@gmail.com (M.A.d.R.B.F.L.); ciubotaru@iom.cnr.it (C.D.C.); 3 Department of Physics, University of Trieste, Via A. Valerio 2, 34127 Trieste, Italy

**Keywords:** urine analysis, digital holographic microscopy, bacteria detection, urinary tract infection, sample screening

## Abstract

Urinary tract infections are among the most frequent infectious diseases and require screening a great amount of urine samples from patients. However, a high percentage of samples result as negative after urine culture plate tests (CPTs), demanding a simple and fast preliminary technique to screen out the negative samples. We propose a digital holographic microscopy (DHM) method to inspect fresh urine samples flowing in a glass capillary for 3 min, recording holograms at 2 frames per second. After digital reconstruction, bacteria, white and red blood cells, epithelial cells and crystals were identified and counted, and the samples were classified as negative or positive according to clinical cutoff values. Taking the CPT as reference, we processed 180 urine samples and compared the results with those of urine flow cytometry (UFC). Using standard evaluation metrics for our screening test, we found a similar performance for DHM and UFC, indicating DHM as a suitable and fast screening technique retaining several advantages. As a benefit of DHM, the technique is label-free and does not require sample preparation. Moreover, the phase and amplitude images of the cells and other particles present in urine are digitally recorded and can serve for further investigation afterwards.

## 1. Introduction

Urinary tract infections (UTIs) are among the most common infections, with major impacts on the individual health and social cost of the healthcare system [1]. UTIs include the presence of bacteria in urine and hence the detection and measurement of bacteria concentration represent important means to support diagnosis based on clinical symptoms. To evaluate UTIs, the urine culture plate test (CPT) is the gold standard method [2]. However, the CPT has the disadvantages that it usually requires more than 24 h and it is costly. Faster and more cost-effective approaches, based on dipstick analysis, are not satisfactory in terms of specificity and sensitivity [2,3]. Urine flow cytometry (UFC) is an attractive alternative for diagnosis of UTIs, leading to a reduction in cultures and antibiotics [4,5,6,7]. One of the most diffused UFC systems on the market is the Sysmex urine particle analyzer UF-1000i, which uses fluorescence flow cytometry technology, offering two separate channels: one for bacteria and one for sediment particles [8,9]. The sample, after being prepared by mixing the urine with a diluent and staining solution at a specific ration, is delivered to a flow cell using a sheath flow technique to produce a single-object stream which is intercepted by a laser beam. Fluorescence forward- and side-scattered light signals are detected and analyzed. Recently, a new flow cytometer (UF-5000) proposed by Sysmex [10] included an additional depolarized side-scattered light for better discrimination of red blood cells and crystals and the ability to differentiate Gram-negative bacteria.

Traditional flow cytometers and flow microscopes, as presented above, provide single-cell fluorescence signal and 1D or 2D spatial information on the objects suspended in liquid jet. Conversely, digital holographic microscopy (DHM) is a phase imaging technique that provides phase information (optical path difference, OPD) and hence 3D information of the sample [11]. The hologram is first recorded on a digital camera as the interference pattern between the reference beam and the object beam is diffracted by the sample. The recorded hologram is then numerically processed to reconstruct the amplitude and the OPD of the object beam. Since the OPD is proportional to the product between the refractive index of the material and the geometrical path of the light, it provides 3D information. Cell parameters as area, volume, perimeter, nucleus volume and 3D shape of the cell and its nucleus can be measured by DHM and monitored in time. These characteristics allowed the use of DHM to study cell tomography, dynamics and growth [11,12,13,14,15], cancer prognosis [16], malaria analysis [17], sickle cell disease [18] or therapeutic efficiency evaluation [19]. An extensive critical review on DHM, the main principles of its operation and current biomedical applications can be found in [20]. Several DHM instruments with different optical configurations have been commercially proposed for living cell studies. Some examples include Ovizio [21], LyncéeTec [22] and Phase Holographic Imaging PHI AB [23]. For instance, the Ovizio system has been recently employed for label-free leukemia detection by using in-flow DHM to extract cell parameters as the optical volume and height [21]. The technique employs sample preparation and 2D hydrodynamic focusing.

Different versions of custom DHM have been reported in the literature in the last years for new applications in biomedicine. Thus, quantitative phase imaging (QPI) has been proposed to extract spatial signatures of cancer cells by discriminating the different stages of oncogenesis [24]. A set of 15 parameters, derived from the cellular 3D morphology and texture have been extracted for suspended healthy and cancer cells (without flow). These quantitative phase-based parameters were useful to discriminate cancer cells. Since QPI allows accurate measurement of single-cell dry mass, it was undertaken to improve the diagnostic accuracy of malignancy in urine cytology [25]. QPI of unstained samples on ThinPrep urine cytology slides from 28 patients with four categories of cytological diagnosis (negative, atypical, suspicious and positive for malignancy) were analyzed. Nuclear/cell dry mass, their entropy and nucleus-to-cell mass ratio were calculated for several hundred cells for each patient and were then correlated with follow-up diagnoses.

Despite these promising DHM applications, to our knowledge, there are no studies reporting the use of DHM for urine analysis in flowing samples. In this paper we introduce an in-flow DHM method to analyze urine samples from patients without any sample preparation. The sample flows in a capillary and a DHM movie is recorded for 3 min. Bacteria and other particles present in urine are detected from numerical reconstruction of the holographic movie using the size, shape and OPD value as parameters. Samples are classified in positives and negatives according to standard clinical cutoff values for bacteria, white blood cells, fungi and epithelial cells. The presence of red blood cells and crystals is also discussed. DHM results are compared with the results obtained with UF-1000i and CPT for the same samples, showing a similar performance as UF-1000i, when compared to CPT as a reference.

## 2. Materials and Methods

### 2.1. In-Flow DHM

**Figure 1:**
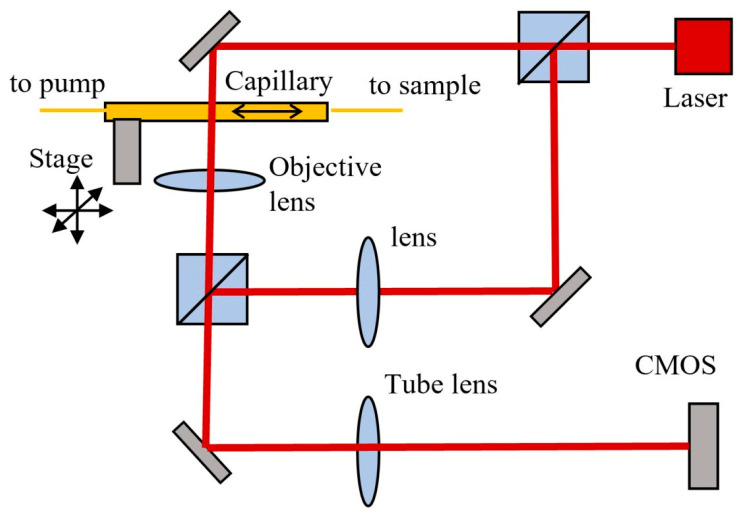
DHM Setup: laser beam (red) is split in two and recombined by two cube beam splitters, which are being directed to the CMOS; the urine is flowing in the capillary and imaged by the objective lens and tube lens on CMOS.

The measurement setup is composed of a custom digital holographic microscope (DHM) based on a Mach-Zender interferometer, a capillary positioned on a xyz microstage, fluidics tubing and a syringe pump (Smart Syringe Pump, Parker, Hendrik-Ido-Ambacht, The Netherlands), as shown schematically in Figure 1.

**Figure 2:**
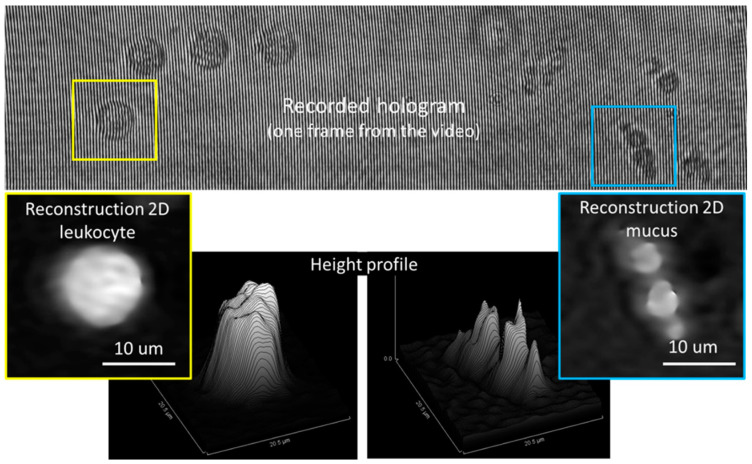
Example of recorded hologram (**top** image) and reconstructed phase images of a leukocyte (**bottom-left** yellow inset) and mucus (**bottom-right** blue inset) with their respective height profiles.

A laser beam with wavelength λ = 632.8 nm, 3 mW (HNL 050R, Thorlabs Inc., Newton, NJ, USA) is split in two beams by a cube beam-splitter (50/50). The first beam illuminates the sample, which is imaged by an objective lens f = 4.51 mm, NA = 0.55 (C230TME-B, Thorlabs) and the tube lens f = 200 mm (TTL200, Thorlabs) on the sCMOS sensor (CS2100M, Thorlabs) with 45× magnification. The second (reference) beam is directed through an identical lens as the objective lens and then deviated by a second beam-splitter to merge the object beam and obtain an interference pattern on the CMOS. Rotation of the second cube beam splitter allows the adjustment of the angle between the reference and object beams. This was adjusted to have an inter-fringe of about 7–8 pixels on the interference pattern, which allows a good spatial separation of the diffraction orders in the Fourier space, as required for an optimal reconstruction. A glass capillary (inner/outer diameter 0.8/1.0 mm, length 200 mm) and fluidics tubing are connected to the pump and the flow rate of the liquid in the circuit is computer controlled. The capillary position was adjusted to have the focal plane of the objective lens at about 5–7 μm from the bottom of the inner wall. A maximum volume of 1 mL liquid can be introduced in the circuit from input/output reservoirs. The liquid sample can be flowed at controlled flow rates between 10 and 100 μL/min for video recording with rates between 1 and 30 frames per second (fps) and 2 ms exposure time/frame, resulting in a holographic movie. Each video frame has 1920 × 1080 pixels, 16 bits depth/pixel. After video recording, the phase and amplitude functions are numerically reconstructed using custom Matlab code (Mathworks) and then the phase function is processed using image processing plugins Fiji (version 1.53t) [26] to facilitate bacteria localization. Brightness/contrast adjustment is applied to the phase image to help the operator localize small bright spots as region of interest (ROI). The size, shape and phase profile are then analyzed for each ROI in the original phase image to confirm the presence of bacteria within the ROI. An example of a recorded hologram (frame) and phase images obtained by numerical reconstruction are shown in Figure 2. One can notice the curved shape of the fringes in the hologram, due to the cylindrical beam shaping by the capillary intercepting the object beam. As background hologram in the reconstruction step, we used the median over all the frames of the holographic movie. The phase images provide useful information about the height (OPD) which allows particle identification.

### 2.2. Sample Handling

The study was performed on 180 urine samples from anonymous patients, kindly provided by a regional general hospital, from 9 June to 15 September 2022. All the samples were first analyzed in the hospital microbiological laboratory by flow cytometry technique using the commercial urine particle analyzer UF-1000i instrument [3], reporting the concentrations of bacteria (BACT), white blood cells (WBL), fungi (F), epithelial and squamous cells (ESC) [27,28,29,30,31]. 

DHM measurements were performed in the same day 3–4 h after the flow cytometry, to avoid bacterial growth in the urine samples. Before DHM analysis, each sample was plated on agar medium using a standard CPT protocol and used as a reference to evaluate the results of the other two techniques. Before loading into DHM instrument, the transparence of each sample was screened by a photo-densitometer (Densimat, Biomerieux, Marcy-l’Étoile, France), and samples exceeding an optical density OD > 1 were discarded and analyzed separately after 1:100 dilution in physiological solution.

### 2.3. Culture Plate Test (CPT)

After homogenizing by manual mixing, 10 µL from each urine sample was streaked on a chromogenic agar plate (ChromID^®^ CPS^®^ Elite, REF. 418284, Biomerieux, Marcy-l’Étoile, France) by standard loop and incubated for 18–24 h at 35 ± 2 °C. After incubation time, the bacterial colony growth on the culture plate was counted (as CFU/mL and identified for species [32,33,34].

### 2.4. Measured Parameters and Evaluation Criteria

To identify and count the particles present in urine samples, we measured the size, shape, area and the optical path difference (OPD) for each potential object detected after the digital reconstruction of the optical phase function from the recorded video. Image processing for digital reconstruction, segmentation and morphological value calculation was performed using ImageJ plugins and custom Matlab code (Mathworks). The analyzed particles were bacteria (BACT), white blood cells (WBL), fungi (F), red blood cells (RBC), epithelial and squamous cells (ESC) and crystals (C).

A urine sample was considered positive with a bacteria concentration C_BACT > 40.000 CFU/mL [33,34]. The results obtained by UF-1000i and DHM techniques were then compared with respect to CPT positives (Pos Ref) and negatives (Neg Ref), determining the true positives (TP), true negatives (TN), false positives (FP) and false negatives (FN). Moreover, seven evaluation parameters, sensitivity (Sens), specificity (Spec), positive predictive value (PPV), negative predictive value (NPV), true positive ratio (TPR), false positive ratio (FPR) and accuracy (ACC), were calculated as defined in Table 1 [35]. A deeper classification in positive or negative was performed while also analyzing the concentration values for the other particles; cutoff in CFU/mL: WBL (40.000), F (150.000), ESC (30.000), RBC (10.000), C (1.000) [33,34].

## 3. Results and Discussion

### 3.1. In-Flow DHM of Microbeads in a Glass Capillary

To test the feasibility of our system to detect microparticles flowing in a glass capillary, we first used 2 μm diameter microbeads of two different materials (silica and polystyrene), which mimic the size, refractive index and density of Escherichia coli bacteria. In fact, the refractive index *n* = 1.384 for *E. coli* [36] is comparable with that of silica (*n* = 1.457 at λ = 632.8 nm, *ρ* = 2.22 g/cm^3^) while the density *ρ* = 1.1 g/cm^3^ is closer to polystyrene (*n* = 1.578, *ρ* =1.05 g/cm^3^) [37].

**Figure 3:**
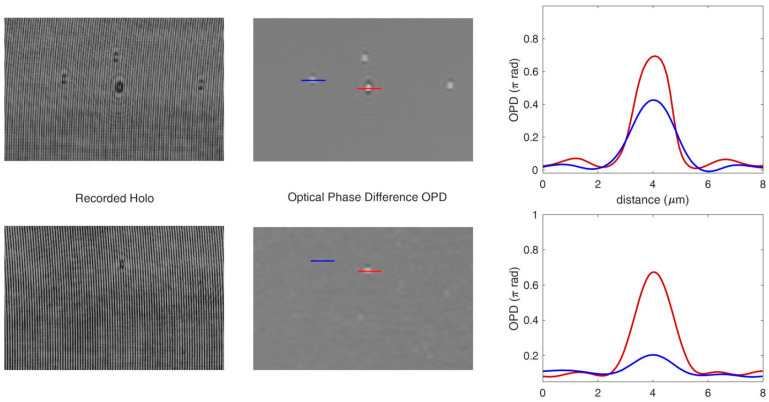
Optical phase difference (OPD) functions reconstructed numerically from recorded holograms of silica (blue) and polystyrene (red) microbeads: 2 μm polystyrene and 2 μm silica beads (top); 2 μm polystyrene and 1 μm silica beads (down).

Colloidal solutions of mixed microbeads in water were prepared at concentration 10^4^ particles/mL. Before loading the sample with beads into the fluidic circuit, this was filled with water from the reservoir, and then a volume of 1 mL of solution was aspired from the sample vial. The beads solution was then pushed by the pump in the capillary (length 200 mm) at a flow rate of Q = 30 μL/min for 5 min to obtain a smooth laminar flow and favorize the beads settling at the bottom of the capillary. This initial step was followed by video recording at 2 fps for 3 min, with the focal plane of the objective at about 5–7 μm above the bottom of the capillary. Using the median over all the recorded holograms as a reference hologram, the amplitude and the optical phase difference (*OPD*) functions were reconstructed numerically (Figure 3). The *OPD*, or the phase delay, is related to the refractive index difference, dn, between the particle and the medium and the geometrical path of the light, l, through the particle [38]:

(1) $$OPD=\frac{2\pi }{\lambda }dn\cdot l$$

Although both silica and polystyrene beads had the same diameter, they could still be distinguished by their refractive indexes. Thus, considering the refractive index of water, *n* = 1.333, the refractive index differences, *dn*, of silica and polystyrene beads are *dn* = 0.124 and *dn* = 0.245, respectively, producing a bigger contrast for polystyrene beads in the phase image. The *OPD* induced by different bead sizes was also investigated by mixing 1 μm silica and 2 μm polystyrene beads in the colloidal solution, showing that 1 μm silica beads also induce a sufficient phase shift to be detectable by their *OPD* (Figure 3).

Note that with a refractive index difference, *dn* = 0.051, a single *Escherichia coli* of 1 μm size induces an *OPD* = 0.16π [rad]. With a camera sensor with 16-bit pixel depth and using half range (2^15^ = 32,768 levels) to represent the *OPD*, the phase image is still significative for detection of bacteria of micron and submicrometric size.

Another question is whether and how can we relate the detected particles to the colloidal solution concentration. To answer this question, we considered the distribution of the particles and related the number of detected particles to the volume of the liquid flowed during the measurement.

Since the microparticles are not uniformly distributed in the capillary volume, the probability for a particle to be found at a given height (*h*) from the bottom of the capillary is ruled by the Boltzmann law [39]:

(2) $$p\left(h\right)=\frac{1}{Z}e^{-\frac{\left(\rho_{p}-\rho_{m}\right)Vh}{KT}}$$

where *ρ_p_* and *ρ_m_* are the mass densities of the particle and the medium, *V* is the particle volume, *K* is the Boltzmann constant, *T* is the temperature and Z the partition function: $Z=\sum e^{-\frac{\left(\rho_{p}-\rho_{m}\right)Vh}{KT}}$.

Since the mass density is high for silica, the particles are located mostly near the bottom of the capillary and the probability of finding them near the focal plane (h < 7 μm) is *p* = 0.98 for both 2 and 1 μm beads. The corresponding probabilities for the polystyrene beads, which are lighter, are *p* = 0.96 for beads of 2 μm diameter and *p* = 0.36 for beads of 1 μm diameter. These probability values indicate the volume near the bottom of the capillary as the best measurement region. If *Nd* is the number of particles detected during the experiment and p the probability of finding the particles in the volume investigated by the objective lens, the total number of particles Nt flowing in the capillary during the experiment will be:

(3) Nt=a· Nd /p

where *a*= [1.1–1.2] is a correction coefficient taking into account the slight perturbations of the laminar flow. This value was established empirically from experiments with beads at a known concentration. Considering the total volume (*Vt*) flowing in capillary during the measurement, the corresponding concentration will be:

(4) C=Nt/Vt=a·Nd/(p·Vt)·1000

in particles/mL. For instance, to have a concentration *C* = 10^4^ particles/mL, the theoretical number of detected particles in a sample of silica beads with 2 μm (or 1 μm) diameter for a flowing volume *Vt* = 90 uL is *Nd* = 800 particles. For polystyrene particles, the number of detected particles, *Nd*, corresponding to a concentration *C* = 10^4^ particles/mL will be *Nd*_2μm_ = 785 and *Nd*_1μm_ = 295 particles, respectively. To check the relation between the concentration, C, and the number of detected particles, Nd, we prepared ten samples of silica beads. We used 2 μm diameter beads at *C* = 10^4^ beads/mL and measured the corresponding Nd for a volume *Vt* = 90 uL flowing in 3 min, obtaining a mean/std value of *Nd*_mean_ = 772/65, which is in agreement with the theoretical expected value. There were about 2.14 beads counted, in average, for each of the 360 recorded frames in the holographic video. These results indicate that our technique can be applied to detect concentrations from 10^3^ to 10^5^ particles/mL, allowing a good discrimination, at least of one order of magnitude in concentration.

To apply the technique to bacteria cells we must consider that the mass density of bacteria is higher than polystyrene, ρ = 1.1 g/cm^3^, and hence the probability of finding 1 μm size bacteria at height *h* < 7 μm is also higher, *p* = 0.59.

### 3.2. In-Flow DHM of Urine Sample in a Glass Capillary

**Figure 4:**
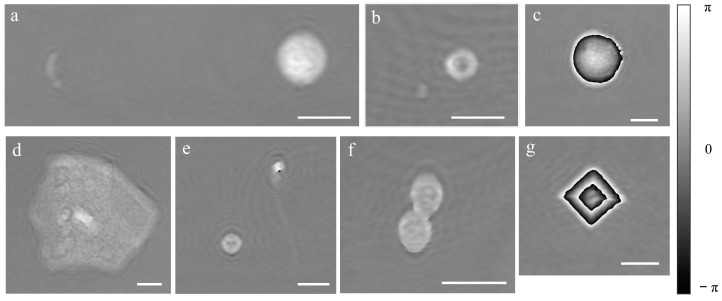
Examples of phase images for different components of the urine samples: (**a**) *Streptococcus* spp. chain (left) and leukocyte (right), (**b**) *Escherichia coli* (left) and red blood cell (right), (**c**) macrophage cell, (**d**) epithelial (squamous) cell, (**e**) red blood cell (down-left) and spermatozoa cell (up-right), (**f**) fungi and (**g**) crystal. Scale bar 10 μm.

Urine samples from patients were handled as described in the “Material and Methods” section. The fluidic circuit was first filled with milliQ water and then the urine sample was loaded by aspiration from the output. A volume of 1 mL was aspired in the pump reservoir and then pushed towards the output at a rate of 30 μL/min for 5 min for flow stabilization, followed by another 3 min for recording. A holographic video was recorded at 2 fps, resulting in a holographic movie of 360 frames for each sample. The OPD was then calculated from a hologram reconstruction. Examples of OPDs obtained for various elements that could be found in different urine samples can be observed in Figure 4 and Video S1. A *Streptococcus* spp. chain is shown together with a white blood cell in Figure 4a, an Escherichia coli together with a red blood cell in Figure 4b, a macrophage in Figure 4c and an epithelial (squamous) cell is reported in Figure 4d. In addition, a red blood cell together with a spermatozoa cell are shown in Figure 4e, fungi in Figure 4f and a crystal in Figure 4g. As one can see, the phase image provides 2D morphological information and an image, but also relevant information on the height and refractive index which can be used to identify the cell. In fact, the gray levels in the images represent the OPD values, in radian. For bacteria and almost all the other cells OPD < 2π rad. For bigger cells or larger refractive index variations, OPD > 2π rad, and the color jumps from white to black (macrophage and crystal). To obtain the real OPD values in these cases, a phase unwrapping procedure is required. However, since the full reconstruction of these types of objects did not affect our analysis, we did not proceed with unwrapping the OPD function.

### 3.3. Bacteriuria Detection and Samples Classification into Positives or Negatives

To test our in-flow DHM technique for detection of bacteria and sample classification, we analyzed 180 fresh urine samples as described in the “Materials and Methods” section. We recorded and reconstructed the holographic movies for each sample as described in the previous section. Then, we processed the OPD function for each sample for the 360 frames/sample containing the phase images. Contrast adjustment followed by shape and size evaluation of the particles in the visual field was used to select bacteria cells and their positions. The presence of bacteria was then confirmed by evaluating the value of the OPD at the respective locations. An OPD value in the range [0.08 π–0.32 π] rad was used to confirm the presence of bacteria. The bacteria were counted in each frame and those bacteria presented in more than one frame were subtracted from the total to obtain the detected number (*Nd*). Following the rationale described in Section 3.1, we used the probability value *p* = 0.59 in Equation (3) to find the total number of bacteria cells *Nt*. For the other cells (WBC, RBC, ESC) we used a higher probability value *p* = 0.88, as derived from the Boltzmann distribution law. Then the concentration was calculated according to Equation (4).

The cutoff value for positive/negative samples was 40.000 CFU/mL [33,34]. With this criterion we detected 133 (73.89%) positive (POS) samples and 47 (26.11%) negative (NEG) samples using the DHM technique. Flow cytometry with UF-1000i found 114 (64.41%) POS and 61 (34.46%) NEG, while the CPT reported 91 (50.56%) POS and 89 (49.44%) NEG (Table 2a, first row). These results show that both DHM and UF-1000i found more POS and less NEG than the CPT. 

Comparing the results obtained by all three techniques, we found a set of 11 samples which were indicated as NEG by CPT but were found POS both by DHM and UF-1000i, i.e., they were false positives for both techniques when compared to CPT. This situation might be explained by a possible antibiotic treatment used by the patients, which inhibits bacteria proliferation and hence the urine sample results as NEG in CPT. Therefore, we also ran the analysis on the pool of data after removing these 11 samples (Table 2a, second row). Taking CPT as reference we calculated the TP, TN, FP and FN for DHM and UF-1000i (Table 2b, first two rows). The percentage of TP and FN for DHM and UF-1000i compared to CPT are similar, while DHM found significantly more FP than UF-1000i. Calculating the sensitivity, specificity and the other five parameters defined in Section 2.4, we found similar values for DHM and UF-1000i when compared to CPT, excepting the specificity and false positive ratio, for which the differences were bigger (Table 2c, first columns).

To evaluate the importance of the presence of other cells in urine, we selected the samples found in the critical range of positivity for bacteriuria, as measured by DHM: 4 × 10^4^ < C_BACT < 6 × 10^4^ CFU/mL. Thus, we found 17 samples in this range and for each of them we analyzed the concentrations of WBL, ESC and F cells. The positivity of the urine sample was confirmed if at least one of the concentration values was within conventional positivity values: C_WBL > 4 × 10^4^ cells/mL, C_ESC > 3 × 10^4^ cells/mL, C_F> 15 × 10^4^ CFU/mL [33,34]. Following this criterion, the positives and negatives for DHM changed from 17 positives and 0 negatives to 5 positives and 12 negatives (Table 2a,b, blue rows). For positives, the number of TP was reduced by 2, while FP was strongly reduced, by 10. For negatives, the number of TN was much increased (by 10) while the FN also slightly increased (by 2). This led to a clear improvement of the values of DHM evaluation parameters compared to CPT (Table 2c, blue column) better approaching the UF-1000i results.

Although the values for the NPV and the TPR (two parameters of most interest when trying to sort out the negative samples) are high (>80%), both methods (DHM and UF-1000i compared to CPT) require further optimization. Moreover, although the FPR is relatively low (<40%), it is still far from the desirable value (<20%) [32] and hence further optimization or cross-checking of the results with other methods are necessary when using DHM or UF-1000i. Investigating a bigger sample volume might enhance the DHM performance, with the cost of the additional time requested for measurement.

DHM also provided information on the presence of RBC, salt/crystals and the type of epithelial cells (cylinders, squamous) in the urine samples. For instance, among the 17 samples selected above there were five samples with a concentration of RBC, C_RBC > 10^4^ cells/mL. Although RBC concentration cannot be correlated with infection (in fact only 1 out of 5 samples was found as FP by DHM vs. CPT), a high concentration of C_RBC might indicate a pathological case, hence providing useful information for clinicians. In addition to RBC presence, three samples were detected with a salt/crystal concentration of C_Crys > 10^3^ particles/mL, which was considered critical. However, only one of these samples was classified as positive, while the other two were negative. Interestingly, although in one of the two negative samples the concentration of crystals and RBC were 8 × 10^4^ particles/mL and 1.1 × 10^4^ cells/mL, respectively, the sample was found negative by all the three techniques (CPT, UF-1000i and DHM) from the bacteriuria point of view. As with the RBC, the presence of a high number of salts/crystals cannot be correlated with infection but it might indicate improper function of the urinary system.

## 4. Conclusions

In the present study, we proposed a new application of DHM to analyze urine samples flowing in a capillary. The measurement was performed in 3 min, flowing the sample at 30 μL/min and acquiring holograms at 2 fps. The reconstruction of the holographic movie allowed us to obtain the phase function (optical path difference, OPD) which, together with the size and shape of the particles, provided the necessary information to detect bacteria and other cells such as WBC, RBC, spermatozoa, epithelial cells and sediments such as crystals and fungi.

To evaluate the performance of DHM as an inspection technique, we performed a screening test on 180 human urine samples and compared the results with those obtained by the standard culture plate test. Moreover, flow cytometry tests were performed on the analyzed samples in the same day by the microbiology laboratory in hospital. The results obtained by DHM and flow cytometry were similar when compared with the CPT, indicating DHM as a simple and fast technique for a preliminary screening of the urine samples.

Better values of the evaluation parameters, compared to CPT, were obtained for DHM by analyzing the presence of other cells in the urine samples at the border of the bacteriuria cutoff, demonstrating the usefulness of the additional information provided by the phase images of the urine components.

The DHM technique is commonly known as a label-free technique, providing an important advantage over the other two techniques: the phase images of all the particles flowing in the field of view are digitally recorded and can be archived and shared for further consultation by different clinicians.

## Figures and Tables

**Table 1:** Evaluation parameters/metrics for positive/negative classification.

Sensitivity %	TP/(TP + FN) × 100
Specificity %	TN/(TN + FP) × 100
Positive predictive value %	TP/(TP + FN) × 100
Negative predictive value %	TN/(TN + FN) × 100
True positive ratio %	TP/Pos Ref × 100
False positive ratio %	FP/Neg Ref × 100
Accuracy %	(TP + TN)/Tot × 100

**Table 2:** Results of the analysis for N = 180 samples measured by DHM, UF-1000i and CPT. (**a**) Positives and negatives as reported by DHM, UF-1000i and CPT, (**b**) True positives (TP), true negatives (TN), false positives (FP), false negatives (FN) for DHM and UF-1000i considering CPT as reference. (**c**) Evaluation parameters in %.

(**a**)											
White row: 180 samples in total, considering bacteriuria Green row: 169 samples in total, considering bacteriuria (11 samples excluded) Blue row: 169 samples in total, considering bacteriuria, WBL, F and ESC											
**Number** **of** **samples**	**DHM**			UF-1000i *			**CPT**				
	POS	NEG	**TOT**	POS	NEG	**TOT**	POS		NEG		**TOT**
	133	47	**180**	114	61	175 *	91		89		**180**
	122	47	**169**	103	61	**164**	91		78		**169**
	110	59	**169**								
(**b**)											
**Number** **of** **samples**	**DHM vs. CPT**				**UF-1000i vs. CPT**						
	TP	TN	FP	FN	TP	TN		FP		FN	
	82	38	51	9	79	52		35		9	
	82	38	40	9	79	52		24		9	
	80	48	30	11							
(**c**)											
**%**			**DHM vs. CPT**					**UF-1000i vs. CPT**			
**Sensitivity**			90.11		90.00	**87.91**		89.77		**89.66**	
**Specificity**			42.70		48.10	**61.54**		59.77		**67.53**	
**Positive Predictive Value**			61.65		66.39	**72.73**		69.30		**75.73**	
**Negative Predictive Value**			80.85		80.85	**81.36**		85.25		**85.25**	
**True Positive Ratio**			90.11		90.00	**87.91**		86.81		**86.67**	
**False Positive Ratio**			57.30		51.90	**38.46**		39.33		**31.65**	
**Accuracy**			66.67		70.41	**75.74**		74.86		**78.78**	

(**a**) * Only 175 of 180 are considered because UF1000i reports were missing for 5 samples.

## Data Availability

Data supporting the reported results is available on request from the corresponding author.

## Supplementary Materials

The following supporting information can be downloaded at: https://www.mdpi.com/article/10.3390/bios13080789/s1, Video S1: In-flow urine DHM.
